# Immunization with Live-Attenuated RHΔ*had2a* Strain Confers Partial Protective Immunity against Acute and Chronic Infection of *Toxoplasma gondii* in Mice

**DOI:** 10.3390/pathogens13020121

**Published:** 2024-01-28

**Authors:** Hai-Sheng Zhang, Hui Cao, Chen-Xu Li, Zhi-Wei Zhang, Meng Wang, Xing-Quan Zhu, Xiao-Nan Zheng

**Affiliations:** 1 Laboratory of Parasitic Diseases, College of Veterinary Medicine, Shanxi Agricultural University, Jinzhong 030801, China; zhs15095372330@126.com (H.-S.Z.); caohui9091@126.com (H.C.); sxaulcx@126.com (C.-X.L.); 2 State Key Laboratory for Animal Disease Control and Prevention, Key Laboratory of Veterinary Parasitology of Gansu Province, Lanzhou Veterinary Research Institute, Chinese Academy of Agricultural Sciences, Lanzhou 730046, China; zhangzhiweicaas1@126.com (Z.-W.Z.); wangmeng02@caas.cn (M.W.)

**Keywords:** *Toxoplasma gondii*, toxoplasmosis, HAD phosphatase, CRISPR-Cas9, live-attenuated strain, vaccine

## Abstract

Toxoplasmosis caused by *Toxoplasma gondii* is an important zoonosis of human and animal health significance. Current chemical therapeutics have side effects, and no commercially available vaccine is licensed for the prevention of toxoplasmosis in humans and most animals. Developing a safe and effective vaccine with long-term protection against *T. gondii* infection is necessary to control toxoplasmosis. HAD2a is a key member of the haloacid dehalogenase (HAD) phosphatase family, which is essential for *T. gondii* daughter budding. However, the role of HAD2a in *T. gondii* virulence remains unknown. In this study, we successfully constructed the *had2a* gene knockout strain in the *T. gondii*-type I RH strain (RHΔ*had2a*) and determined its role in virulence and vaccination. These results demonstrate that HAD2a played an important role in parasite daughter budding and in vitro replication. Knockout of the *had2a* gene attenuated the virulence of the *T. gondii*-type I RH strain. Vaccination with RHΔ*had2a* tachyzoites induced a Th1-biased immune response, provided partial protection against acute *T. gondii* infection in mice by highly virulent tachyzoites of RH and PYS (ToxoDB#9, Chinese I) strains, and conferred strong protection against challenge infection by cysts and oocysts of the less virulent type II Pru strain. These results demonstrate that *T. gondii had2a* is important for its in vitro proliferation and virulence in mice and that RHΔ*had2a* may be used as a candidate strain to generate a multiple gene knockout live-attenuated strain or be collaboratively applied with other live-attenuated strains to confer more effective protection against *T. gondii* infection.

## 1. Introduction

*Toxoplasma gondii* is an obligate intracellular protozoan that infects almost all warm-blooded animals and approximately 25 to 30% of the world’s human population [1,2,3]. Foodborne transmission is the primary route of *T. gondii* infection in humans [4,5]. The consumption of undercooked or raw meat products contaminated with cysts or ingestion of vegetables or water contaminated with oocysts may result in *T. gondii* infection [1,2,3,4,5]. Infection with *T. gondii* severely threatens the health of pregnant women and immunocompromised patients (such as HIV and organ transplant patients) [5,6,7]. Primary *T. gondii* infection during pregnancy may cause premature delivery, stillbirth, congenital abnormalities, developmental disabilities, growth retardation and meningitis in fetuses [5,6,8,9]. Although significant advances have been achieved in studies of *T. gondii* biology and epidemiology, current toxoplasmosis treatment, mainly pharmacotherapy, can only manage acute and reactivated infections without the clearing of *T. gondii* tissue cysts [2,5,8].

Preventing *T. gondii* infection or clearing latent infection may be achieved by vaccination. Toxovax, a live-attenuated vaccine derived from *T. gondii* S48 tachyzoites and licensed in a few countries, has been used in sheep against toxoplasmosis, especially preventing miscarriage in pregnant sheep [10]. However, this licensed vaccine has some application defects, such as a relatively short shelf life, uncomplete block to congenital infection and a possible reversion to a virulent phenotype [8]. Significant toxoplasmosis vaccine development has been explored in the last few decades, such as live-attenuated strains, DNA vaccines, epitope vaccines, exosome-based, carbohydrate-based and nanoparticle-based vaccination [8,11,12]. With the application of the CRISPR-Cas9 gene-editing tool in *T. gondii* research, several knockout strains of important *T. gondii* genes have been tested in vaccination studies in mice [8,13,14]. These virulence-attenuated vaccine strains include RHΔ*tkl1* [13], RHΔ*gra17Δnpt1* [15], RHΔ*ompdc*Δ*uprt* [16], ME49Δ*cdpk3* [17] and PruΔ*cdpk2* [18], which have provided efficient protection against acute, chronic, and congenital infection.

HAD2a (TGGT1_289910), which possesses a haloacid dehalogenase (HAD) phosphatase domain, is essential for *T. gondii* daughter budding [19]. Conditional knockout of the *had2a* gene leads to incomplete cytokinesis, conjoined daughters, and disrupted proliferation [19]. However, the role of HAD2a in *T. gondii* virulence remains unknown. The in vitro replication defect caused by the conditional knockout of the *had2a* gene indicates that the *had2a* gene mutant strain might have replication defect and attenuated virulence, suggesting a potential role as a live-attenuated vaccine. Here, we successfully constructed the *had2a* gene knockout strain RHΔ*had2a* and characterized the virulence of RHΔ*had2a* in mice. The immunization results revealed that RHΔ*had2a* vaccination significantly induced specific anti-*T. gondii* antibodies and conferred partial protection against *T. gondii* acute and chronic infection. Our findings suggest that HAD2a is important for *T. gondii* virulence and RHΔ*had2a* can be used as a parental strain to generate an attenuated multiple gene knockout vaccine strain.

## 2. Materials and Methods

### 2.1. Animals and Ethics Statement

The female Kunming mice (6–8 weeks old) used in the experiment were purchased from the Laboratory Animal Center of Lanzhou Veterinary Research Institute, Chinese Academy of Agricultural Sciences (Lanzhou, China). To reduce the impact of stress response on the experiment, mice were placed in a temperature-controlled room with a 12 h light/dark cycle and provided with sufficient sterile water and food for free intake. The study was approved by Animal Ethics Committee of Lanzhou Veterinary Research Institute, Chinese Academy of Agricultural Sciences (Approval no. 2020-022). Every effort was made to reduce any suffering of the animals.

### 2.2. Parasite Culture

*Toxoplasma gondii* tachyzoites of the parental RH∆*ku80* (referred as RH) stain, PYS (ToxoDB#9, Chinese I) strain and gene mutant strains were maintained in confluent monolayers of human foreskin fibroblasts (HFFs, ATCC SCRC-1041^TM^) maintained in DMEM supplemented with 2% fetal bovine serum (FBS, Gibco, Auckland, New Zealand), 10 mM HEPES (pH 7.2, Solarbio, Beijing, China), 100 U/mL of penicillin (Solarbio, China) and 100 μg/mL of streptomycin (Solarbio, China) as described previously [20,21]. For further study, the tachyzoites were isolated and purified by 27-gauge needles (BD Medical, Franklin Lake, WI, USA) and Millipore filters (Merck-Millipore, Darmstadt, Germany) with a pore size of 5 µm. Tachyzoites were counted by hemocytometer measurement and diluted to required number of tachyzoites in 200 μL PBS or DMEM [22].

### 2.3. Construction of had2a Gene Knockout Strain

The CRISPR-Cas9 mediated homologous gene recombination was used to delete the *had2a* gene in the wild-type RH strain [23]. The CRISPR plasmid pSAG1::CAS9-U6::Sg HAD2a and homologous dihydrofolate reductase (DHFR) drug-selective plasmid were constructed. To generate the *had2a* gene knockout CRISPR plasmid pSAG1::CAS9-U6::Sg HAD2a, the SgRNA of *had2a* gene was used to replace the SgRNA of the *uracil phosphoribosyl transferase* (*uprt*) gene in the pSAG1::CAS9-U6::SgUPRT template plasmid [24]. The 5′ and 3′ homologous arms of the *had2a* gene (1000~1300 bp) were fused to the DHFR fragment and the pUC19 backbone fragment to construct the homologous DHFR-selective plasmid [25]. The sequence-validated plasmid was used as a template to amplify the homologous DHFR fragment. To construct the *had2a* gene knockout strain (RHΔ*had2a*), the purified homologous drug-selective cassette and sequencing-validated CRISPR plasmid were co-transfected into the wild-type RH tachyzoites. After pyrimethamine selection, single clones of RHΔ*had2a* obtained by modified limiting dilution were confirmed by designed PCRs [20]. The primers used are listed in Table 1.

### 2.4. Immunofluorescence Assay (IFA)

To visualize the effect of *had2a* gene deletion on parasite daughter budding, the purified RH and RHΔ*had2a* tachyzoites were used to infect the HFF monolayers. After fixed with 4% paraformaldehyde for 20 min, the samples were permeabilized with 0.2% Triton X-100 for 15 min and incubated with polyclonal rabbit anti-IMC1 (1:500, available in our lab) [20] and goat anti-rabbit IgG (H+L) conjugated with Alexa Fluor 488 (1:500, Thermo Fisher Scientific, Waltham, MA, USA) for 1 h at 37 °C, each of which was followed by five washes with PBS [26]. A Leica confocal microscope system (TCS SP8, Leica, Wetzlar, Germany) was used to image the samples.

### 2.5. Plaque Assay

Confluent HFF monolayers grown in 12-well plates (Thermo Fisher Scientific, USA) were infected with 300 freshly egressed tachyzoites of wild-type RH or mutant RHΔ*had2a* strains for 7 days. After the culture medium was removed, samples were washed with PBS, fixed with 4% paraformaldehyde for 20 min and stained with 0.2% crystal violet for 20 min, which was followed by two washes with PBS [25]. Three independent replicates were performed. The plaque size and number were analyzed with the ImageJ software version 1.53a.

### 2.6. Intracellular Replication Assay

Freshly egressed tachyzoites (1 × 10^5^) of RHΔ*had2a* or RH strain were used to infect the confluent HFF monolayers cultured in 12-well plates for 1 h prior to washing the uninvaded tachyzoites. The number of tachyzoites in at least 100 parasitophorous vacuoles of samples was counted using IFA after 24 h incubation, in which mouse anti-SAG1 (1:500, Thermo Fisher Scientific, USA) and goat-anti mouse conjugated with Alexa Fluor 488 were used to visualize intracellular parasites [27].

### 2.7. Virulence Assessment in Mice

Female Kunming mice were randomly divided into six groups (10 mice/group). Different doses of RH (1 × 10^2^) or RHΔ*had2a* (1 × 10^2^, 1 × 10^3^, 1 × 10^4^, 1 × 10^5^, 1 × 10^6^) tachyzoites diluted in 200 μL PBS were used to infect mice intraperitoneally. During 30 days of infection, the survival and clinical signs of all infected mice group were observed twice daily [28]. Mice reaching a humane endpoint were euthanized immediately.

### 2.8. Antibodies Assessment in Immunized Mice

Kunming mice were intraperitoneally immunized with 5 × 10^4^ RHΔ*had2a* tachyzoites, which was diluted in 200 μL PBS and quantified by plaque assay to ensure its number and viability used in vaccination. Naive mice were intraperitoneally injected with 200 μL PBS. Serum samples of the naive and immunized mice (six mice per group) were collected at 30 days post-immunization. To detect whether anti-*T. gondii* antibodies were induced, subclass antibodies IgG1 and IgG2a along with total IgG were determined using ELISA [13,15].

### 2.9. Protection of RHΔhad2a Immunization against T. gondii Infection

Thirty days post-vaccination with 5 × 10^4^ RHΔ*had2a* tachyzoites, 1 × 10^3^ *T. gondii* tachyzoites of the wild-type RH strain or PYS strain were used to intraperitoneally infect each of the immunized mice and naive mice (ten mice per group). For chronic infection, the naive and immunized mice (ten mice per group) were orally administrated with 20 Pru cysts or 50 Pru oocysts. The Pru cysts were collected and purified from the brain tissues of Kunming mice infected with cysts according to previous descriptions [13,29], whereas the Pru oocysts were collected and purified from feces of kittens orally infected with cysts [13,30,31]. The survival time and toxoplasmosis clinical signs of all infected mice group were observed twice daily. Mice were euthanized immediately when reaching the humane endpoint. Brain cysts of all chronically survived mice were counted as previously [20,25]. For the detection of brain cyst burden, the brains of the mice that survived for 30 days post-chronic challenge were collected, homogenized, and used to count brain cyst number, as described previously [15].

### 2.10. Statistical Analysis

All data obtained from three independent replicates were represented as means ± standard deviations (SD). Group difference was analyzed by a two-tailed, unpaired Student’s *t* test using GraphPad Prism version 9.0. Difference was considered significant when the *p* value < 0.05.

## 3. Results

### 3.1. Successful Construction of RHΔhad2a

**Figure 1:**
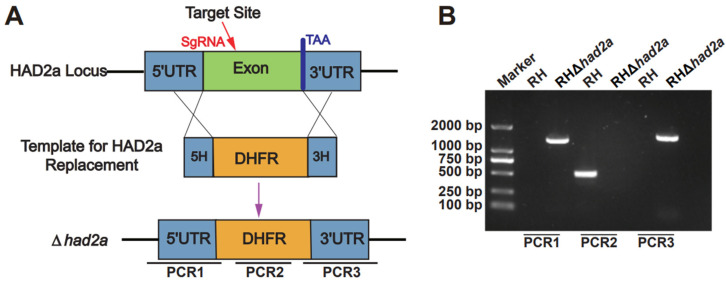
Construction and validation of RHΔ*had2a*. (**A**) Schematic illustration of *had2a* gene deletion by CRISPR-Cas9 mediated homologous gene replacement. (**B**) PCRs of RHΔ*had2a*. Recombinant homologous fragments of 5′ and 3′ in RHΔ*had2a* were detected by PCR1 and PCR3. Deletion of *had2a* gene was detected by PCR2.

To investigate the role of HAD2a in *T. gondii* virulence, the *had2a* gene was knocked out in the *T. gondii*-type I RH strain by replacing the *had2a* gene with a homologous DHFR fragment using CRISPR-Cas9 (Figure 1A). The successful construction of gene knockout strain RHΔ*had2a* was confirmed by PCRs. In PCR1 and PCR3, recombinant 5′ and 3′ homologous fragments (~1300 bp) were amplified in the RHΔ*had2a* strain but not in the RH strain (Figure 1B), validating the successful insertion of 5′ and 3′ homologous fragments in RHΔ*had2a*. The small *had2a* coding gene fragment was amplified in PCR2 of the RH strain but not in PCR2 of the RHΔ*had2a* strain (Figure 1B), confirming the disruption of the *had2a* gen in RHΔ*had2a*. These results demonstrated that *had2a* gene knockout strain RHΔ*had2a* was successfully generated by CRISPR-Cas9 mediated homologous recombination.

### 3.2. Knockout of had2a Gene Severely Attenuated T. gondii Virulence in Mice

**Figure 2:**
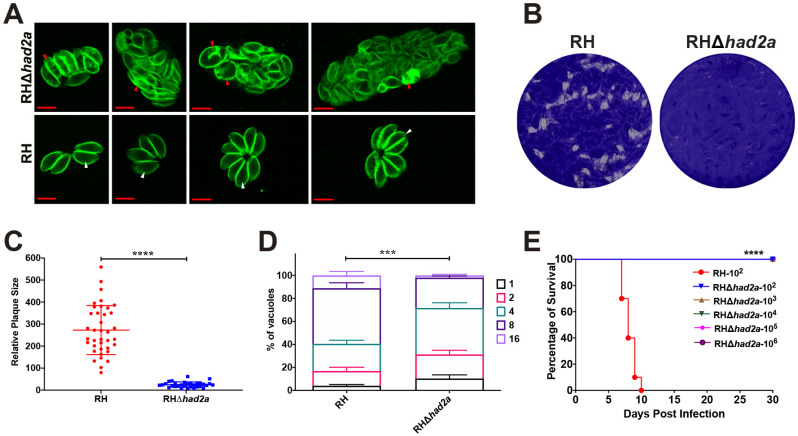
Knockout of *had2a* affected *T. gondii* daughter budding, replication, and virulence. (**A**) Division defect of intracellular RHΔ*had2a* dividing parasites. Wild-type RH parasites show normal daughter parasite budding (white arrows), while some RHΔ*had2a* parasites show the aberrant morphology of intercellular parasites and improper segregation of daughter cells (red arrows). (**B**–**D**) Replication defect of RHΔ*had2a* parasites in vitro. Plaques comparison of wild-type RH and RHΔ*had2a* parasites (**B**). Relative size and number of plaques were significantly affected by *had2a* gene deletion (**** *p* < 0.0001) (**C**). (**D**) Intracellular replication of RH and RHΔ*had2a* parasites at 24 h post infection of HFFs determined in three independent experiments. RHΔ*had2a* parasites had a slower replication compared to wild-type RH strain (*** *p* < 0.001). (**E**) Attenuated virulence of RHΔ*had2a* parasites. All mice infected with different doses of RHΔ*had2a* tachyzoites survived, while all mice infected with wild-type RH tachyzoites were sacrificed (**** *p* < 0.0001).

To assess the effect of *had2a* disruption on the replication and virulence of *T. gondii*, we performed IFA, in vitro plaque and in vivo virulence assays using RHΔ*had2a* and wild-type RH strains. In IFA, a division defect was observed in RHΔ*had2a* (Figure 2A). Some intravacuolar tachyzoites of RHΔ*had2a* were enlarged without daughter budding or the separation of dividing tachyzoites, lacking the rosette appearance of wild-type RH parasites. These results confirmed the role of HAD2a in parasite daughter budding as previously described [19]. In an in vitro plaque assay, the same dose of RHΔ*had2a* or RH tachyzoites were used to infect the HFF monolayer. After 7 days of incubation, the HFF samples were stained with crystal violet. The results showed that knockout of the *had2a* gene caused a significant defect in the plaque-forming ability (Figure 2B,C). The in vitro parasite replication rates were also monitored 24 h post infection. The deletion of *had2a* led to a significant decrease in the replication of *T. gondii* type I strain (Figure 2D). The significant defect of RHΔ*had2a* in plaque-forming ability and replication efficacy demonstrates that HAD2a plays an important role in the in vitro proliferation of *T. gondii*.

To test whether HAD2a is important for *T. gondii* virulence, different doses of RHΔ*had2a* or RH tachyzoites were used to infect Kunming mice. All mice infected with different doses (10^2^, 10^3^, 10^4^, 10^5^, 10^6^) of RHΔ*had2a* tachyzoites survived without any clinical symptoms of toxoplasmosis, whereas mice infected with 10^2^ tachyzoites of the wild-type RH strain were sacrificed within 10 days post-infection (Figure 2E). These results suggest that HAD2a is important for *T. gondii* virulence and that RHΔ*had2a* may be a potential candidate for developing anti-*T. gondii* live-attenuated vaccine.

### 3.3. RHΔhad2a Vaccination Induces a Th1-Dominated Immune Response

**Figure 3:**
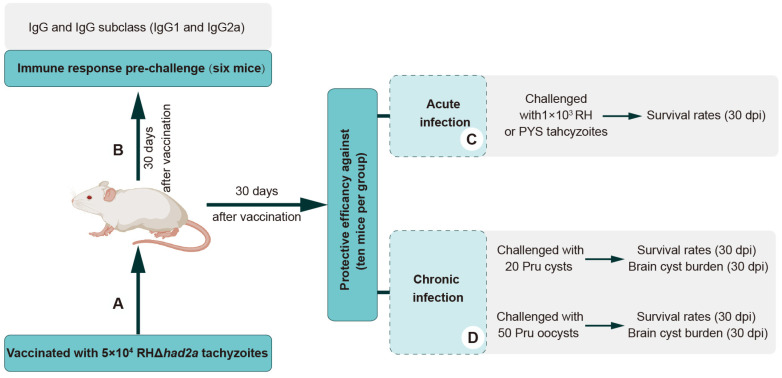
Schematic illustration of the study design of RHΔ*had2a* vaccination. Experimental overview of Kunming mice vaccination with 5 × 10^4^ RHΔ*had2a* tachyzoites (**A**), and determination of anti-*T. gondii* antibodies of vaccinated mice prior to challenge (**B**) at acute (**C**) and chronic stages of infection (**D**).

**Figure 4:**
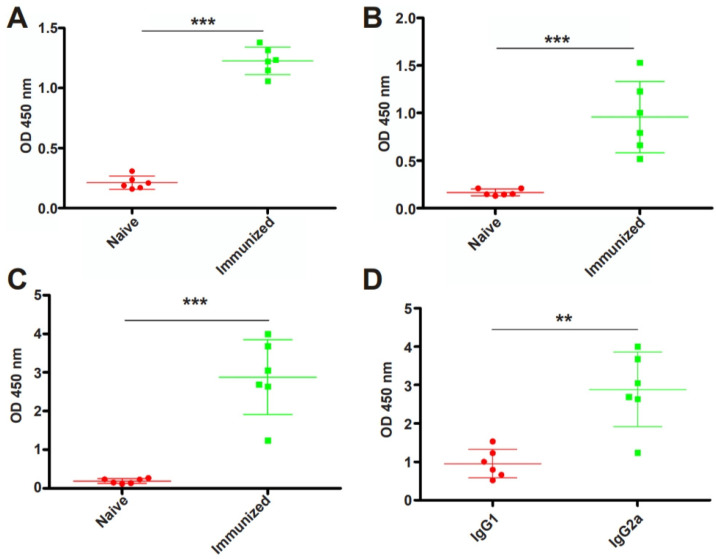
Immunization with RHΔ*had2a* induced a significant increased level of anti-*T. gondii*-specific antibodies. Mice were vaccinated with 5 × 10^4^ RHΔ*had2a* tachyzoites for 30 days. Total IgG (**A**), IgG1 (**B**) and IgG2a (**C**) in sera of the naive and immunized mice (*** *p* < 0.001). Comparison of IgG1 and IgG2a levels in immunized mice (** *p* < 0.01) (**D**).

To examine whether RHΔ*had2a* vaccination induces the production of anti-*T. gondii*-specific IgG, Kunming mice were immunized with 5 × 10^4^ RHΔ*had2a* tachyzoites (Figure 3A) [25]. The sera of RHΔ*had2a*-vaccinated and unvaccinated mice were isolated at 30 days post-infection. The total IgG, subclass antibodies IgG1 and IgG2a were determined by ELISA (Figure 3B). These results showed that the sera obtained from RHΔ*had2a*-vaccinated mice had significantly higher levels of IgG, IgG1 and IgG2a compared with the sera of non-vaccinated mice (Figure 4A–C). The IgG2a level was higher than the IgG1 level in the sera of RHΔ*had2a*-vaccinated mice (Figure 4D). These results illuminated that RHΔ*had2a* vaccination induced a Th1-dominated immune response and that RHΔ*had2a* has the potential to be a live-attenuated vaccine candidate.

### 3.4. Vaccination of RHΔhad2a Provides Partial Protection in Mice against Acute Infection of Virulent Strains

**Figure 5:**
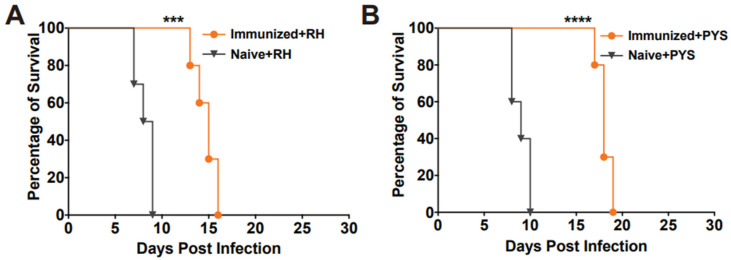
RHΔ*had2a* immunization significantly prolonged the survival time of mice with acute *T. gondii* infection. The naive and immunized mice were challenged with 1 × 10^3^ tachyzoites of type I RH strain (**A**) or PYS (ToxoDB#9, Chinese I) strain (**B**) 30 days after immunization (*** *p* < 0.001, **** *p* < 0.0001). The survival time of the vaccinated mice was significantly longer compared to that of the naive mice.

To assess the protective efficacy of RHΔ*had2a* vaccination against tachyzoite infection, Kunming mice were immunized with 5 × 10^4^ RHΔ*had2a* tachyzoites (Figure 3A) [25]. After 30 days post-immunization, the vaccinated mice and unvaccinated mice were infected by 10^3^ wild-type RH or PYS tachyzoites (Figure 3C). The results showed that all unvaccinated mice were sacrificed within 10 days when challenged with RH or PYS tachyzoites (Figure 5A,B). Although the vaccinated mice were also sacrificed, the survival time was significantly prolonged in the vaccinated group (sacrificed within 18 days) (Figure 5A,B). These results indicate that immunization of the live-attenuated RHΔ*had2a* confers partial protection against acute *T. gondii* infection by virulent strains.

### 3.5. RHΔhad2a Vaccination Confers Partial Protection against Chronic Infection

**Figure 6:**
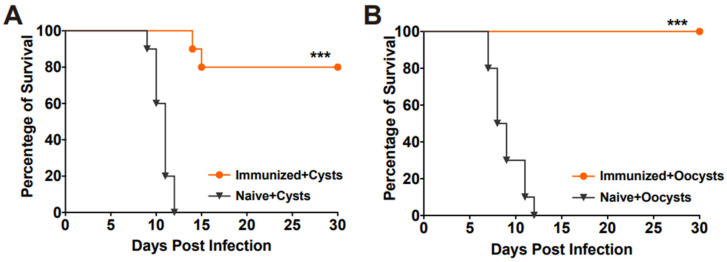
Vaccination with RHΔ*had2a* protected mice from chronic *T. gondii* infection. Survival time of the naive and immunized mice orally infected with 20 cysts (**A**) or 50 oocysts (**B**) of type II *T. gondii* Pru strain, 30 days after immunization with RHΔ*had2a* tachyzoites (*** *p* < 0.001). Survival percentages of the vaccinated mice were significantly higher than that of the naive mice.

To evaluate the protective efficacy of RHΔ*had2a* vaccination against chronic infection, mice were orally challenged with 20 Pru cysts or 50 Pru oocysts after 30 days post-vaccination (Figure 3D). The results showed that all unimmunized mice were sacrificed during the chronic stage of challenge infection by cysts or oocysts of the Pru strain (Figure 6A,B), whereas 80% of the vaccinated mice in the cysts-challenged group and 100% of the vaccinated mice in the oocysts-challenged group survived (Figure 6A,B). At 30 days post-challenge, the brain cyst numbers of the surviving mice were determined. In immunized groups, two out of ten mice in the Pru cysts-challenged group were sacrificed, and brain cysts were detected in only three out of the eight surviving mice (median = 45 ± 32). In the oocysts-challenged group, only three out of ten surviving mice had brain cysts (median = 20 ± 22). The numbers of brain cysts in the surviving vaccinated mice were significantly lower than that previously reported [15,25]. These results suggest that RHΔ*had2a* vaccination induced partial protection against chronic *T. gondii* infection.

## 4. Discussion

Toxoplasmosis caused by *T. gondii* infection is a significant public health threat to humans and animals [5,32]. Due to its elaborate immune evasion system and complex life cycle of *T. gondii*, it is difficult to prevent and treat toxoplasmosis [5,32]. Although the combined use of pyrimethamine and sulfadiazine is the primary therapeutic regimen, failure of treatment happened quite often [8]. Side effects of current therapeutics may occur in treated patients [33]. Vaccination of humans and animals with effective vaccines would reduce chemical therapeutic consequences and reliance, and it offers a better alternative for the efficient and long-term control of zoonotic toxoplasmosis [8,34].

HAD2a is essential for *T. gondii* daughter budding, which is an important part of the parasite lytic cycle [19]. In this study, we successfully knocked out the *had2a* gene in the RH strain and constructed an RHΔ*had2a* mutant strain. These results of IFA, plaque assay and intravacuolar replication assay demonstrated the important role of HAD2a in daughter budding, the separation of dividing tachyzoites and intracellular proliferation, which was consistent with a previous study [19]. We used 10^2^, 10^3^, 10^4^, 10^5^, and 10^6^ RHΔ*had2a* tachyzoites and 10^2^ RH tachyzoites to infect Kunming mice and monitored the survival rates of the mice. All infectious doses of RHΔ*had2a* tachyzoites were not lethal to mice and caused no clinical symptoms of toxoplasmosis, suggesting that the deletion of *had2a* attenuated the virulence of the RH strain, indicating the possible application of RHΔ*had2a* as a toxoplasmosis vaccine candidate. Infection of mice with the wild-type RH strain can cause histopathological changes in the liver, spleen, brain and lung of the infected mice [35]. Thus, future studies should examine the histopathological changes in the mice infected with RHΔ*had2a* tachyzoites to evaluate its safety in mice.

*Toxoplasma gondii* infection induces a complex protective immunity, including the innate immune response, adaptive immune response, and humoral immunity [36,37]. In this study, we found that RHΔ*had2a* vaccination induced a significant increase in anti-*T. gondii* specific antibodies, with significantly higher IgG2a levels than IgG1 levels, indicating that the vaccination of mice with RHΔ*had2a* provokes a Th1-biased immunity. The increased IgG induced by RHΔ*had2a* immunization may opsonize *T. gondii* by phagocytosis, inhibiting the attachment of the parasites to host cells and blocking the parasite invasion to exert their protective effect against *T. gondii* infection [38]. The host immunity is primarily dependent on T helper 1 (Th1) cell-mediated immunity [8,36,37]. High levels of interleukin-12 (IL-12) and interferon-γ (IFN-γ) are critical for the clearance of tachyzoites during acute infection, and it is essential for the formation of tissue cysts and sustainment of latent infection [8,36,37,39]. Whether RHΔ*had2a* vaccination induces the production of specific cytokines, such as IFN-γ, TNF-a, IL-12 or MCP-1, requires further studies. In addition, the determination of cytokines and antiparasitic factors, such as reactive nitrogen species and oxygen species, in mice infected with different doses of tachyzoites may help to understand whether the possible protective effect of a live-attenuated strain is dependent on the inoculated dose.

Tachyzoites and bradyzoites are the main infectious stages of *T. gondii*’s asexual reproductive cycle. Rapidly dividing tachyzoites can disseminate to enormous and distant host tissues and provoke significant immune responses [5]. Host immune defense and drug application can limit tachyzoite growth; however, some tachyzoites overcoming these challenges transform into slowly replicating bradyzoites [5]. Cyst walls protect slowly dividing bradyzoites within tissue cysts to remain dormant in hosts by evading host immune responses, facilitating the establishment of long-term persistent infection [5,40]. *Toxoplasma gondii* infection commonly occurs in humans via the oral consumption of water, vegetables, fruits contaminated with *T. gondii* oocysts or undercooked meat containing *T. gondii* tissue cysts [41]. Thus, challenge infection with tachyzoites, cysts and oocysts after the immunization of mice with live-attenuated *T. gondii* vaccine strains is important in anti-*T. gondii* vaccine research.

In this study, we found that the survival rate was significantly higher in vaccinated mice challenged with Pru cysts compared with that of unvaccinated mice. Remarkably, RHΔ*had2a* vaccination protected all mice from Pru oocyst infection. The brain cyst number of vaccinated mice challenged with Pru cysts and oocysts was significantly decreased comparing to previous data [15,25]. However, *T. gondii* might be detected in other organs of the infected mice, in which no brain cyst was detected [17]. In addition, the *T. gondii* load in vaccinated mice should be assessed before and after re-infection, in future studies, to rule out the residual infection and to verify that parasites are effectively eliminated by immunization.

Comparing to the naive group, mice vaccinated with RHΔ*had2a* tachyzoites had a significantly longer survival time when challenged with tachyzoites of the wild-type I RH strain and PYS strain (ToxoDB#9). These results demonstrate that vaccination with the RHΔ*had2a* strain conferred partial protection against acute infection and partially prevented the establishment of parasite infection in mice challenged with the Pru strain. Unlike RHΔ*tkl1* [13], ME49Δ*α-amy* [14] and RHΔ*gra17Δnpt1* [15], RHΔ*had2a* did not fully protect mice from the virulent RH and PYS strains. Thus, RHΔ*had2a* may not be individually applied as an effective live-attenuated vaccine. However, RHΔ*had2a* may be applied to generate a multiple-attenuated knockout vaccine strain in further studies. One limitation of RHΔ*had2a* constructed in this study is that it exhibits resistance to pyrimethamine, which is one of the primary drugs for treating toxoplasmosis. Thus, RHΔ*had2a* should not be used for humans.

## 5. Conclusions

The findings of the present study demonstrates that the deletion of *had2a* suppressed the daughter budding and in vitro growth, and it attenuated the virulence of the *T. gondii*-type I RH strain. A single vaccination intraperitoneally of 5 × 10^4^ RHΔ*had2a* tachyzoites induces a Th1-skewed immune response in mice. Immunization of the RHΔ*had2a* mutant strain provides a significantly longer survival time for mice against acute infection by highly virulent tachyzoites compared to the naive mice, partial protection against chronic infection by Pru cysts, and strong protection against Pru oocysts. These findings show that RHΔ*had2a* could be a promising parent strain to generate a double- or triple- gene knockout mutant strain, or it could be cooperatively applied with another live-attenuated strain as live-attenuated vaccines.

## Figures and Tables

**Table 1:** Primers used in this study.

Primer	Sequence (5′-3′)	Use
SgHAD2a-KO-F	GCAATGTTGAAGGCGAAGCC	SgRNA of CRISPR plasmid for HAD2a knockout
SgRNA-R	AACTTGACATCCCCATTTAC	Construct the CRISPR plasmid for deleting HAD2a
SgHAD2a-KO-F	GCAATGTTGAAGGCGAAGCCGTTTTAGAGCTAGAAATAGC	Construct the CRISPR plasmid for deleting HAD2a
U5-HAD2a-F	GGTTTTCCCAGTCACGACGTTAACCGTCTGTGCCGGTATTG	Amplify the 5′ homologous arm of HAD2a
U5-HAD2a-R	GGATTTACAGCCTGGCGAAGCTTTACAAGCGGGACTTACCTTCA	Amplify the 5′ homologous arm of HAD2a
U3-HAD2a-F	CTATGCACTTGCAGGATGAATTCTAGAGGCACCGTTGTCAGCA	Amplify the 3′ homologous arm of HAD2a
U3-HAD2a-R	GAGCGGATAACAATTTCACAAAACGGCTACACTGCGTGGT	Amplify the 3′ homologous arms of HAD2a
DHFR-F	AAGCTTCGCCAGGCTGTAAATCC	Amplify the DHFR fragment
DHFR-R	GAATTCATCCTGCAAGTGCATAG	Amplify the DHFR fragment
pUC19-F	TGTGAAATTGTTATCCGCTC	Amplify the pUC19 fragment
pUC19-R	AACGTCGTGACTGGGAAAACC	Amplify the pUC19 fragment
HAD2a-DHFR-F	AACCGTCTGTGCCGGTATTG	Amplify the 5H-DHFR-3H fragments
HAD2a-DHFR-R	AAACGGCTACACTGCGTGGT	Amplify the 5H-DHFR-3H fragments
PCR1-HAD2a-F	AATCAACGTAGCGCTGCTTGCTTC	Detect the insertion of 5′ homologous fragment in PCR1
PCR1-HAD2a-R	GCCAAAGTAGAAAGGAATTAGCAT	Detect the insertion of 5′ homologous fragment in PCR1
PCR2-HAD2a-F	GACAGCACAGAGAAACCCCT	Detect the deletion of HAD2a in PCR2
PCR2-HAD2a-R	GGTGGTGATTGTCACGAACG	Detect the deletion of HAD2a in PCR2
PCR3-HAD2a-F	TGACGCAGATGTGCGTGTATCCAC	Detect the insertion of 3′ homologous fragment in PCR3
PCR3-HAD2a-R	CACACTGAGTCAGTAGCGTGTACA	Detect the insertion of 3′ homologous fragment in PCR3

## Data Availability

The original contributions presented in this study are included in the article. Further inquiries can be directed to the corresponding authors.
